# Trends in Low-Density Lipoprotein Cholesterol Goal Achievement in High Risk United States Adults: Longitudinal Findings from the 1999–2008 National Health and Nutrition Examination Surveys

**DOI:** 10.1371/journal.pone.0059309

**Published:** 2013-04-02

**Authors:** Matthew C. Tattersall, Ronald E. Gangnon, Kunal N. Karmali, Michael W. Cullen, James H. Stein, Jon G. Keevil

**Affiliations:** 1 Division of Cardiovascular Medicine, Department of Medicine, University of Wisconsin School of Medicine and Public Health, Madison, Wisconsin, United States of America; 2 Department of Population Health Sciences, University of Wisconsin School of Medicine and Public Health, Madison, Wisconsin, United States of America; 3 Division of Cardiovascular Diseases, Department of Medicine, Mayo Clinic, Rochester, Minnesota, United States of America; Universidade de São Paulo, Brazil

## Abstract

**Background:**

Previous studies have demonstrated gaps in achievement of low-density lipoprotein-cholesterol (LDL-C) goals among U.S. individuals at high cardiovascular disease risk; however, recent studies in selected populations indicate improvements.

**Objective:**

We sought to define the longitudinal trends in achieving LDL-C goals among high-risk United States adults from 1999–2008.

*Methods* We analyzed five sequential population-based cross-sectional National Health and Nutrition Examination Surveys 1999–2008, which included 18,656 participants aged 20–79 years. We calculated rates of LDL-C goal achievement and treatment in the high-risk population.

**Results:**

The prevalence of high-risk individuals increased from 13% to 15.5% (p = 0.046). Achievement of LDL-C <100 mg/dL increased from 24% to 50.4% (p<0.0001) in the high-risk population with similar findings in subgroups with (27% to 64.8% p<0.0001) and without (21.8% to 43.7%, p<0.0001) coronary heart disease (CHD). Achievement of LDL-C <70 mg/dL improved from 2.4% to 17% (p<0.0001) in high-risk individuals and subgroups with (3.4% to 21.4%, p<0.0001) and without (1.7% to 14.9%, p<0.0001) CHD. The proportion with LDL-C ≥130 mg/dL and not on lipid medications decreased from 29.4% to 18% (p = 0.0002), with similar findings among CHD (25% to 11.9% p = 0.0013) and non-CHD (35.8% to 20.8% p<0.0001) subgroups.

**Conclusion:**

The proportions of the U.S. high-risk population achieving LDL-C <100 mg/dL and <70 mg/dL increased over the last decade. With 65% of the CHD subpopulation achieving an LDL-C <100 mg/dL in the most recent survey, U.S. LDL-C goal achievement exceeds previous reports and approximates rates achieved in highly selected patient cohorts.

## Introduction

LDL-C reduction is a central component of coronary heart disease (CHD) risk reduction [1]. Over the past two decades numerous clinical trials have demonstrated that lowering LDL-C reduces cardiovascular risk [2]–[11]. Individuals at highest risk of a cardiovascular event derive the greatest benefit from lowering of LDL-C and achievement of LDL-C goals [12]. In the United States, clinical guidelines have been modified to reflect the importance of LDL-C goal achievement in high-risk individuals. In 1993, the National Cholesterol Education Program’s Adult Treatment Panel (ATP) II [13] recommended an LDL-C goal of <100 mg/dL for patients with CHD. In 2001, the ATP III added CHD “equivalent” conditions to the population appropriate for an LDL-C of <100 mg/dL [1]. In 2004, an ATP III update presented an optional high-risk goal of an LDL-C <70 mg/dL [12]. Despite the importance of LDL-C reduction, an achievement gap in U.S. individuals at high cardiovascular risk has been described [14]–[15]. One year after the ATP III recommendations to treat those at high risk to an LDL-C <100 mg/dL, only 32% of the high-risk population in the U.S. met this goal [14]. Over the past decade there has been an increase in the availability of potent lipid-lowering therapies and increasing evidence supporting the benefits of LDL-C reduction [16]. With increasing knowledge, education, and treatment options the previously described gap in LDL-C goal achievement in high-risk individuals may have improved. In 2005, 62% of U.S. CHD patients in a very select physician group achieved an LDL-C <100 mg/dL [17]. A recent report analyzed the prevalence of high LDL-C levels across different risk levels in the National Health and Nutrition Examination Surveys (NHANES) 1999–2006 and found the prevalence of high LDL-C in the U.S. population across each risk category to be improving over the study period [18]. The longitudinal trends in U.S. LDL-C goal achievement at both the ATP III LDL-C goal of <100 mg/dL and the 2004 optional LDL-C goal of <70 mg/dL among the high risk subgroups over the past decade have not been described. Defining LDL-C goal achievement among the high-risk population and respective subgroups offers an opportunity to examine treatment gaps and identify patient populations to target with preventative therapies. The purpose of our analysis was to describe the longitudinal changes in the prevalence of LDL-C goal achievement in high-risk individuals from 1999–2008 in the U.S. population.

## Methods

The National Center for Health Statistics performs the National Health and Nutrition Examination Survey (NHANES) in two-year increments to define the health and nutritional status of the United States population. The NHANES utilizes complex, stratified, multistage sampling techniques based on demographic and geographical data, assigning subjects a weight such that the sum represents a statistical model of the entire civilian non-institutionalized United States population. Methods involve identification of primary sampling units, within which, clusters of households are identified with each person in the household screened for demographic characteristics. The NHANES database has been used to develop national health standards, [19] assess disease prevalence, [20]–[23] identify risk factors for disease development and management, [24]–[25] and assess the health of the nation [26]–[27]. Detailed information on NHANES data collection is published and available at http://www.cdc.gov/nchs/nhanes.htm.

### Inclusions/Exclusions

We analyzed five NHANES surveys: 1999–2000, 2001–2002, 2003–2004, 2005–2006, and 2007–2008. Included participants were ages 20–79 years and met the ATP III definition of “high risk” (see below). We excluded participants who were pregnant, received chemotherapy within 4 weeks of the exam, lacked complete lipid data including (missing total cholesterol, high density lipoprotein levels, LDL-C, or triglycerides) or blood pressure data or had triglyceride measurements ≥400 mg/dL. (Table 1).

**Table 1:** Inclusions/Exclusions.

	NHANES Survey Years				
	1999–2000	2001–2002	2003–2004	2005–2006	2007–2008
	Participants	Participants	Participants	Participants	Participants
Total MEC† population	9282	10,477	9643	9950	9762
<20 years old	4838	5450	4901	5177	4055
>79 years old	322	402	449	358	389
Total MEC ages 20–79 years	4122	4625	4293	4415	5318
Triglycerides ≥400 mg/dL	104	138	95	157	218
Missing blood pressure data	78	142	148	133	161
Missing Lipid data	252	276	248	261	349
Pregnant	259	323	199	325	57
Current chemotherapy*	28				
Peripheral vascular disease#	43	64	59		
Included population	3358	3682	3544	3539	4533

* Variable only collected in 1999–2000.

† MEC Mobile Exam Center.

# Peripheral Vascular Disease only collected in 99–00, 01–02, 03–04.

### High Risk Criteria

We defined “high risk” according to U.S. ATP III guidelines [28] that included those participants with known CHD or one or more CHD risk equivalent conditions including diabetes mellitus, cerebrovascular disease, or the presence of two or more ATP III risk factors and a Framingham risk score ≥ 20% [28]. CHD was defined in the NHANES dataset by self-reported history of myocardial infarction, CHD or angina pectoris. Diabetes mellitus was defined in the NHANES dataset by self-reported history of diabetes or of taking insulin and/or oral hypoglycemic medications. Cerebrovascular disease was defined in the NHANES dataset by self-reported history of a stroke. The number of ATP III risk factors were summed: age (men ≥45 years or women ≥55 years), hypertension (blood pressure ≥140/90 mmHg or use of antihypertensive medication), tobacco use (smoked 100+ lifetime cigarettes and currently smoking), family history of early CHD (defined in NHANES as subjects reporting a heart attack or angina in grandparents, parents or siblings <50 years of age) and high-density lipoprotein cholesterol (HDL-C) <40 mg/dL. An HDL-C ≥60 mg/dL led to subtraction of one risk factor. Framingham risk scores were calculated using formulas provided by the Framingham study (Available at: http://www.framinghamheartstudy.org/risk/hrdcoronary.html).

### Lipid Goal Achievement

Low-density lipoprotein cholesterol (LDL-C) was calculated using the Friedewald equation, regardless of fasting status [14], [29]–[30]. Weighted mean LDL-C concentrations were calculated for those at high-risk and for the subsets with and without CHD. We evaluated achievement of both the ATP III LDL-C goal of <100 mg/dL and the 2004 optional LDL-C goal of <70 mg/dL. To determine those participants who remained eligible for pharmacotherapy initiation or intensification we evaluated the high-risk subset with an LDL-C ≥130 mg/dL. We divided this group into those with and without CHD, and further divided both groups based on medication use.

### Statistical Analysis

The NHANES data sets from 1999–2008 were downloaded and imported into Microsoft Excel (version 11.2 for Macintosh, Microsoft Corp, Redmond, WA) and into SAS version 9.2 (SAS Institute, Cary, NC). Appendix S1 describes the list of NHANES item codes and descriptions. To account for the complex survey design of NHANES, SAS PROC SURVEYMEANS was used to calculate standard errors using the Taylor Linearization method [31]. The 95% confidence intervals for estimated population parameters, p-values for differences in parameters between adjacent surveys and p-values for tests of trend over time were calculated using the Wald method. A two-tailed p-value of less than 0.05 was considered to be significant.

## Results

### Prevalence of High-Risk Conditions

The prevalence of high-risk participants in the entire eligible population increased from 13.0% in 1999–2000 to 15.5% in 2007–2008 (p for trend 0.046) (Table 2). The prevalence of the high-risk subgroup with CHD did not significantly change over the time period of the five surveys (range 4.9% to 6.4% p = 0.33). The subgroup of high-risk participants without CHD increased from 7.5% in 1999–2000 to 10.6% in 2007–2008 (p for trend = 0.002). The prevalence trends of the diabetes mellitus subgroup increased from 5.6% to 8.6% (p for trend = <0.0001) and the cerebrovascular disease subgroup increased from 1.8% to 2.7% (p for trend = 0.032) over the five surveys. The subgroup of high risk with two or more risk factors and a Framingham risk score ≥20% (range 2.2% to 3.3% p = 0.85) did not change significantly.

**Table 2:** Prevalence of High Risk Conditions in Analyzed Populations NHANES 1999–2008.

NHANES Survey Years						
	1999–2000	2001–2002	2003–2004	2005–2006	2007–2008	P value for trend*
High Risk	13.0%	13.2%	15.8%	14.0%	15.5%	0.046
	(11–15.1%)	(11.9–14.6%)	(13.8–17.9%)	(12.5–15.6%)	(13.4–17.6%)	
High Risk with CHD	5.5%	5.4%	6.4%	5.4%	4.9%	0.33
	(4.7–6.4%)	(4.3–6.5%)	(4.8–8.1%)	(4.5–6.3%)	(4.2–5.6%)	
High Risk without CHD	7.5%	7.8%	9.4%	8.6%	10.6%	0.002
	(5.9–9.1)	(6.6–9%)	(8.4–10.4%)	(7.5–9.8%)	(8.9–12.2%)	
High Risk with Diabetes Mellitus	5.6%	6.3%	7.5%	7.8%	8.6%	<0.0001
	(4.6–6.6%)	(5.3–7.3%)	(6.2–8.8%)	(6.6–9%)	(7.1–10.2%)	
High Risk with stroke	1.8%	1.9%	2.5%	2.3%	2.7%	0.032
	(1.1–2.4%)	(1.3–2.5%)	(1.7–3.3%)	(1.6–3.1%)	(2–3.4%)	
High Risk with +2 risk factors and FRS>20%	3.2%	2.6%	3.1%	2.2%	3.3%	0.85
	(2.5–3.9%)	(2.2–3.1%)	(2.5–3.7%)	(1.6–2.7%)	(2.5–4.1%)	

(95% CI).

* p-value for test for trend over time.

CHD = coronary heart disease, FRS = Framingham Risk Score.

### LDL-C Levels in High-Risk Individuals

The mean LDL-C levels of the entire high-risk population and its subgroups with and without CHD significantly decreased over the time period of the five surveys (Table 3). The mean LDL-C in the 1999–2000 survey was 128.1 mg/dL (3.3 mmol/L) (95% CI, 122.8–133.4 mg/dL); this decreased to 106.5 mg/dL (2.8 mmol/L) (95% CI, 102.9–110.1 mg/dL) in 2007–2008 (p<0.0001). In the high-risk subgroup with CHD, the mean LDL-C in 1999–2000 was 123.2 mg/dL (3.2 mmol/L) (95% CI, 117.3–129.1 mg/dL), this level decreased to 98.3 mg/dL (2.5 mmol/L) (95% CI, 92.8–103.7 mg/dL) in 2007–2008 (p<0.0001). In the subgroup of high-risk participants without CHD, the mean LDL-C decreased from 131.7 mg/dL (3.4 mmol/L) (95% CI, 125.4–138 mg/dL) in 1999–2000 to 110.3 mg/dL (2.9 mmol/L) (95% CI, 105.3 –115.3 mg/dL), in the 2007–2008 survey (p<0.0001).

**Table 3:** Population LDL-C Means and Goal Achievement.

	NHANES Survey Years									
	1999–2000	2001–2002		2003–2004		2005–2006		2007–2008		
Mean LDL-C	Mean LDL-C(mg/dL)*	Mean LDL-C(mg/dL)*	P value†	Mean LDL-C(mg/dL)*	P value†	Mean LDL-C(mg/dL)*	P value†	Mean LDL-C(mg/dL)*	P value†	P for trend‡
All High Risk	128.1	120.2	0.0092	122.7	0.3623	107.1	<0.0001	106.5	0.8415	<0.0001
	(122.8–133.4)	(116.4–124)		(118.3–127.1)		(101.8–112.4)		(102.9–110.1)		
CHD subset	123.2	111.7	0.0107	118.9	0.0910	100.5	<0.0001	98.3	0.6439	<0.0001
	(117.3–129.1)	(104.1–119.3)		(113.9–123.9)		(92–109)		(92.8–103.7)		
Non CHD subset	131.7	126.1	0.115	125.3	0.8524	111.2	0.0025	110.3	0.8022	<0.0001
	(125.4–138)	(121.9–130.4)		(117.2–133.5)		(105.4–117)		(105.3–115.3)		
**LDL-C Goal Achievements: Proportion of High Risk achieving LDL-C Goals**										
**High Risk Population**	**Proportion**	**Proportion**	**P value** †	**Proportion**	**P value** †	**Proportion**	**P value** †	**Proportion**	**P value** †	**P for trend** ‡
<100 mg/dL	24%	32.8%	0.005	32.1%	0.837	49%	<0.0001	50.4%	0.712	<0.0001
		(27.3–38.2%)		(27.4–36.7%)		(42.8–55.1%)		(45–55.7%)		
<70 mg/dL	2.4%	7.1%	<0.0001	6.9%	0.869	16.8%	<0.0001	17%	0.933	<0.0001
	(0.9–3.9%)	(5.4–8.8%)		(4.1–9.6%)		(12.5–21.1%)		(14.3–19.7%)		
≥130 mg/dL	44.4%	33.4%	0.0007	37.8%	0.178	25.1%	0.0004	23.8%	0.667	<0.0001
	(39.2.–49.6%)	(28.8–38%)		(32.5–43.2%)		(19.7–30.6%)		(20.2–27.4%)		
**Proportion of High Risk and Coronary Heart Disease Achieving LDL-C Goals**										
<100 mg/dL	27.0%	42.0%	0.014	34.5%	0.141	55.6%	<0.0001	64.8%	0.045	<0.0001
	(17.7–36.3%)	(32.8–51.3%)		(28.7–40.3%)		(48.9–62.3%)		(57.7–71.9%)		
<70 mg/dL	3.4%	11.3%	0.0001	6.8%	0.101	21.7%	<0.0001	21.4%	0.946	<0.0001
	(0.6–6.1%)	(7.8–14.7%)		(2.2–11.4%)		(15.3–28.2)		(14.6–28.2%)		
≥130 mg/dL	37.2%	26.3%	0.018	33%	0.206	19.0%	0.018	18.0%	0.851	<0.0001
	(30.2–44.2%)	(19.4–33.2%)		(24.1–41.9%)		(10–28%)		(12.2–23.9%)		
**Proportion of High Risk and No Coronary Heart Disease Achieving LDL-C Goals**										
<100 mg/dL	21.8%	26.3%	0.197	30.4%	0.367	44.8%	0.004	43.7%	0.248	<0.0001
	(16.9–26.6%)	(20.5–32.2%)		(22.8–38%)		(37.6–52.1%)		(36.6–50.7%)		
<70 mg/dL	1.7%	4.2%	0.048	6.9%	0.133	13.7%	0.011	14.9%	0.962	<0.0001
	(0.2–3.2%)	(1.9–6.5%)		(3.9–9.9%)		(8.9–18.5%)		(11.6–18.2%)		
≥130 mg/dL	49.7.7%	38.3%	0.007	41.1%	0.554	29%	0.015	26.5%	0.414	<0.0001
	(42.8–56.6%)	(32.5–44%)		(32.6–49.7%)		(22.6–35.3%)		(21.7–31.3%)		
**Proportion with both LDL-C ≥130 mg/dL & Not on lipid lowering pharmacotherapy**										
High Risk	35.1%	25.9%	0.0033	28.8%	0.2793	15.5%	<0.0001	18%	0.2475	0.0002
	(29.4–40.9%)	(22.5–29.4%)		(24.3–33.2%)		(12.6–18.4%)		(14.5–21.5%)		
CHD subset	25%	15.7%	0.0371	23.9%	0.0549	11.7%	0.0024	11.9%	0.9616	0.0013
	(17.3–32.6%)	(9.9–21.4%)		(16.8–30.9%)		(7–16.5%)		(6.7–17.2%)		
Non-CHD Subset	42.6%	33%	0.0096	32.2%	0.8085	17.9%	0.0004	20.8%	0.4142	<0.0001
	(35.8–49.4%)	(28.9–37.1%)		(25.8–38.5%)		(12.2–23.6%)		(15.7–26%)		

* To convert mg/dL to mmol/L multiply value by 0.0259.

† p value compared to the previous survey.

‡ p value for test for trend over time.

### Achievement of LDL-C <100 mg/dL in High-Risk Participants

Achievement of LDL-C <100 mg/dL (<2.6 mmol/L) in high-risk participants increased from 24% in 1999–2000 to 50.4% in 2007–2008 (p for trend <0.0001, Table 3). Achievement of LDL-C <100 mg/dL for the subgroup with CHD increased from 27.0% to 64.8% (p for trend <0.0001); high-risk participants without CHD also increased, from 21.8% to 43.7% (p for trend <0.001) [Table 3]. In the 2007–2008 survey, achievement of LDL-C <100 mg/dL in the CHD subset (64.8%) was significantly greater than in the subset without CHD (43.7%, p<0.0001).

### Achievement of LDL-C <70 mg/dL in High-Risk Participants

From the 1999–2000 to the 2007–2008 survey, the proportion of high-risk participants achieving an LDL-C <70 mg/dL (<1.8 mmol/L) increased from 2.4% to 17.0% (p<0.0001). The subgroup of high-risk participants with CHD that achieved an LDL-C <70 mg/dL increased from 3.4% to 21.4% (p<0.0001); high-risk participants without CHD that achieved an LDL-C <70 mg/dL also increased from 1.7% to 14.9% (p<0.0001). In the 2007–2008 survey, achievement of LDL-C <70 mg/dL did not differ significantly between the subgroups with CHD (21.4%) and without CHD (14.9%, p = 0.066).

### High-Risk Participants with LDL-C ≥130 mg/dL and Not on Lipid Medications

The proportion of high-risk participants with LDL-C ≥130 mg/dL (≥3.4 mmol/L) decreased from 44.4% to 23.8% (p<0.0001) from 1999–2000 to 2007–2008. In the subset of participants with CHD, the proportion decreased from 37.2% to 18% (p<0.0001); in the high-risk subset without CHD the proportion decreased from 49.7% to 26.5% (p<0.0001). Overall, there was a significant reduction in the number of untreated high risk with LDL-C >130 mg/dL. In 1999–2000, 35.1% of the high-risk population with an LDL-C ≥130 mg/dL was not on lipid medication. This proportion decreased to 18% in the 2007–2008 survey (p = 0.0002). In 1999–2000, 25% of participants with CHD were not on lipid medications and had an LDL-C ≥130 mg/dL. This increased to 11.9% in 2007–2008 (p = 0.0013). In the subgroup of high-risk participants without CHD, 42.6% were not on lipid medication and had an LDL-C ≥130 mg/dL in 1999–2000. This proportion increased to 20.8% in 2007–2008 (p<0.0001).

## Discussion

### Primary Findings

This study describes U.S. improvements in LDL-C goal achievement in high cardiovascular risk populations over the last decade. Improvements were observed in participants at high risk, both with and without CHD; and at three LDL-C thresholds: <100 mg/dL, <70 mg/dL, and ≥130 mg/dL not on lipid medications.

### Historical Context of LDL-C Goal Achievement in CHD

Studies from the 1980s in the U.S. demonstrated improvements in LDL-C goal achievement in the subset of the high-risk population with CHD. Analyses of the NHANES III survey described LDL-C goal achievement rates between 16.6%–18.0% in individuals with CHD [32]–[34]. A survey of primary clinicians performed in 1993 found that only 14% of the high-risk population achieved the recommended LDL-C goal of less than 100 mg/dL [35]. A comparison of the 1999–2000 and 2001–2002 NHANES survey demonstrated improved achievement of LDL-C <100 mg/dL from 27% to 41% [14]. In 2004, the NCEP Evaluation ProjecT Utilizing Novel E-Technology (NEPTUNE) II, a nationwide survey that recruited from the top 26% statin-prescribing physicians evaluated 4,885 patients with dyslipidemia and reported that 57% of all high-risk patients and 62% of CHD patients achieved LDL-C levels below 100 mg/dL [17]. In 2006–2007, The multinational Lipid Treatment Assessment Project (L-TAP) 2 study evaluated 9,955 dyslipidemic patients on lipid-lowering therapy. Of the 5,930 high-risk individuals with CHD, 82% were on statin therapy and 67% reached their country’s respective goal LDL-C [36]. While these studies provided optimism that achievement of LDL-C goals was improving, they were performed in highly selective clinician or patient cohorts.

This study, in contrast, measures LDL-C goal achievement levels in an U.S. representative high-risk population, providing a comparison for historical cohort studies of LDL-C goal achievement. Improvements in achieving an LDL-C <100 mg/dL goal appeared to occur in plateaus following the release of the ATP III guideline in 2001 [28] and ATP III update guidelines in 2004 [12]. Due to the cross-sectional nature of the study it is not possible to determine causality; however, release of guidelines may be one explanation for the timing of these improvements. Our study provides optimism that LDL-C goal achievement trends in the United States are improving and are consistent with the high goal achievement rates seen in highly selective cohorts.

### CHD Compared with Non-CHD High Risk

LDL-C goal achievement improved in both the “high risk with CHD” subgroup and the “high risk without CHD” subgroup over the time period of the five surveys (p<0.0001 for both). Furthermore, the subgroup of “high risk with CHD” had significantly higher rates of LDL-C <100 mg/dL (p<0.0001) and lower rates of LDL-C ≥130 mg/dL (p = 0.02) compared with the “high risk without CHD” subgroup in the 2007–2008 survey. The “high risk without CHD” subset had a higher rate of LDL-C ≥130 mg/dL and not on lipid medications (20.8%) compared with the “high risk with CHD” subset (11.9%, p = 0.0099). The observed differences in LDL-C goal achievement and lipid medication use between the CHD and non-CHD subsets are likely the result of multiple causes. The high risk without CHD population in this analysis included those with diabetes mellitus, the presence of two or more ATP III risk factors and a Framingham risk score ≥ 20% or cerebrovascular disease. The lipid goal achievement gap in diabetic patients has been well described in the literature and represents numerous factors from failure to treat to the difficulty of clinical inertia in clinical practice. [37]–[39] The LDL-C achievement gap in individuals at high risk by Framingham risk score alone may be due to under recognition. Cardiovascular risk score calculation is low within primary care practices, with one report indicating cardiovascular risk calculation rates of ∼17% in primary care offices [40]. Thus, the failure to treat this group with two or more risk factors and a Framingham risk score may be the result of not appropriately identifying patients at risk. The lack of LDL-C goal achievement in those patients high risk secondary to cerebrovascular disease may also represent a combination lack of knowledge of lipid goals and clinical inertia. The importance of use of lipid lowering medication in patients with CHD is well established with a ∼30% reduction in CHD mortality. The benefits of lipid lowering medications in patients with a history of ischemic stroke is less well established with relative risk reductions ranging from 14–21% [41]–[42]. Whatever the underlying reasons may be for the LDL-C achievement gap between the high risk populations with and without CHD this finding underscores the need to target educational and awareness efforts at high-risk patients without established CHD.

### Limitations/Strengths

This is a serial cross-sectional analysis with limitations inherent to the NHANES survey, including statistical modeling and selection bias. The generalizability of these findings outside the U.S. population may be limited as the NHANES are a population based statistical model of the entire civilian noninstitutionalized United States population. The NHANES questionnaires rely on self-report and may be subject to misunderstanding and recall bias. NHANES does not include incarcerated or institutionalized individuals. Some of the NHANES variables do not match the definitions set forth in the ATP III guidelines precisely so some misclassification may have occurred. For example, the NHANES defines a positive family history of CHD as a parent or grandparent experiencing a myocardial infarction or angina under the age of 50 years without gender differentiation. The ATP III guideline defines a positive family history of CHD as a risk factor if CHD afflicts a first degree male relative under the age of 55 years, or a first degree female relative under the age of 65 years [28]. The NHANES survey does not include testing for aortic aneurysms, a history of aortic surgery and does not include a question regarding claudication. Also, the NHANES survey stopped collecting data on ankle-brachial index after the 2003–2004 survey. This could result in an underestimation of those with peripheral vascular disease. However, those eligible for inclusion with ankle-brachial indices <0.9 represent a very small portion of the high risk population. Responses to questions regarding current use of lipid medications are subject to recall bias and misunderstanding. Furthermore, the cross-sectional design of NHANES makes the treatment effects of the lipid medications impossible to assess.

### Conclusion

Overall, the United States population at high risk for cardiovascular disease experienced significant increases in the proportion of individuals achieving LDL-C goals of <100 mg/dL and <70 mg/dL. There also has been a significant decline in the proportion of the high-risk population with LDL-C ≥130 mg/dL and not on lipid medications.

## Supporting Information

Appendix S1 **NHANES Item ID and Descriptions.**
(DOC)
