# Supplementary material for: Trends in Low-Density Lipoprotein Cholesterol Goal Achievement in High Risk United States Adults: Longitudinal Findings from the 1999–2008 National Health and Nutrition Examination Surveys
Source: PLoS One. 2013 Apr 2;8(4):e59309. doi: 10.1371/journal.pone.0059309 (PMC3615020; doi:10.1371/journal.pone.0059309)
Supplement: Appendix S1 — NHANES Item ID and Descriptions. (DOC) [file pone.0059309.s001.doc]

| **NHANES Item ID** | **Description** |
| --- | --- |
| SEQN | Respondent sequence number |
| RIDSTATR | Interview/Examination Status |
| RIAGEYR | Age at Screening |
| RIAGENDR | Gender |
| WTMEC2YR | Two year MEC sample Exam Weight |
|  |  |
| **Questionnaire** | **Description** |
| BPQ050A | Currently taking prescriptions for high blood pressure |
| DIQ010 | Doctor told you have Diabetes |
| DIQ050 | Taking insulin now? |
| DIQ070 | Take diabetic pills to lower blood sugar? |
| MCQ160C | Ever told you had coronary heart disease? |
| MCQ160D | Ever told you had angina/angina pectoris? |
| MCQ160E | Ever told you had heart attack? |
| MCQ160F | Ever told you had a stroke? |
| SMQ020 | Smoked at least 100 cigarettes in life? |
| SMQ040 | Do you now smoke cigarettes? |
| BPQ100D | Now taking prescribed medicine? (for cholesterol) |
| MCQ250G* | Family History of Early Coronary Disease† |
|  |  |
| **Examination** | **Description** |
| BPXSY | Systolic blood pressure |
| BPXDI | Diastolic blood pressure |
|  |  |
| **Laboratory Assessment** | **Description** |
| LBXTC | Total cholesterol (mg/dL) |
| LBXSTR | Triglycerides (mg/dL) |
| LBDHDL | HDL-C- cholesterol (mg/dL) |

**Appendix A: NHANES Item ID and Descriptions.**

*MCQ300A in 2005-2006 and 2007-2008

†NHANES asked the family history of early coronary artery disease question as follows: “Including living and deceased, were any of biological that is, blood relatives including

grandparents, parents, brothers, sisters ever told by a health professional that they had a

heart attack or angina before the age of 50?”
